# Optical Coherence Tomography in Infectious Keratitis After Femtosecond Keratorefractive Surgery

**DOI:** 10.3390/jcm14041067

**Published:** 2025-02-07

**Authors:** Antonio Leccisotti, Stefania V. Fields, Giuseppe De Bartolo, Christian Crudale, Matteo Posarelli

**Affiliations:** 1 Siena Eye Laser, 53036 Poggibonsi, Italy; stefania.fields@virgilio.it (S.V.F.); giusedebartolo@gmail.com (G.D.B.); christiancrudale@gmail.com (C.C.); mposarelli@gmail.com (M.P.); 2 School of Biomedical Sciences, Ulster University, Coleraine BT52 1SA, UK; 3 Centre for Research in Refractive Surgery, 53035 Siena, Italy; 4 Department of Ophthalmology, Liverpool University, Liverpool L3 5TR, UK

**Keywords:** optical coherence tomography, laser in situ keratomileusis (LASIK), keratorefractive lenticule extraction (KLEx), infectious keratitis

## Abstract

**Objectives**: Anterior Segment Optical coherence tomography (AS–OCT) can help in the diagnosis and treatment of infectious keratitis, but it has not been studied in cases occurring after corneal refractive surgery procedures such as femtosecond laser in situ keratomileusis (FS–LASIK) and keratorefractive lenticule extraction (KLEx). In these procedures, a surgical interface is created, where infections usually start, thus determining a different AS–OCT pattern compared to non–surgical infections, which begin on the corneal surface. **Methods**: We retrospectively reviewed 22,756 eyes of 13,564 patients who underwent FS–LASIK and KLEx at our surgical center. **Results**: Four cases of post–refractive surgery infectious keratitis were included (two after FS–LASIK and two after KLEx), in which the AS–OCT identified an initial infiltrate in the interface, followed by interface inflammation. In one case, after FS–LASIK, interface fluid accumulation occurred. In one case, after KLEx, diffuse interface inflammation led to stromal reabsorption, later compensated by stromal reformation and epithelial hyperplasia, well documented by OCT. **Conclusions**: AS–OCT represents a useful tool for assessing corneal infections after refractive surgery, guiding the treatment, and evaluating the healing process and residual corneal scarring.

## 1. Introduction

Corneal infections after refractive surgery are considered a severe complication due to the presence of a surgical interface that limits the effect of the topical treatment [1]. Incidence of infectious keratitis (IK) has been reported to range between 0.02% and 0.8% after photorefractive keratectomy (PRK) and between 0% and 1.5% after laser in situ keratomileusis (LASIK) [1,2]. Keratorefractive lenticule extraction (KLEx) is a relatively newer procedure with more controversial data found in the literature, but we have recently demonstrated that IK incidence might be higher than LASIK and PRK [3]. IK is considered an ocular emergency and requires prompt management to avoid sight–threatening complications [4]. Diagnosis commonly relies on slit–lamp examination, corneal imaging, and corneal cultures [4,5,6,7], but it can be challenging for many reasons. First, slit–lamp assessment requires expertise, and patients might have discomfort and might not be compliant during the examination [8]. Second, the positive culture rate can range from 30 to 68%, according to many studies previously published worldwide [9,10,11,12,13]. American Society of Cataract and Refractive Surgery (ASCRS) recommends flap lifting to facilitate culture and irrigation [6,14], but positive microbiological results have been found in less than 50% of the cases [15,16,17], and patients can experience worsening of the symptoms after interface irrigation [1,18]. Thus, considering the limitations of the clinical evaluation, efforts have been made to improve corneal infections in clinical practice [8,19]. A corneal biopsy is used in case of infection progression or poor response to treatment, but it is an invasive procedure and can cause corneal perforation, irregular astigmatism, and corneal scarring [20]. Impression cytology can be used to obtain cell samples from the ocular surface, but it is less useful for infections affecting the surgical interface, such as after refractive surgery [21]. Among the imaging modalities available, In Vivo Confocal Microscopy (IVCM) can provide high–quality in vivo images of the cornea at a cellular level [22,23]. Many studies have demonstrated that IVCM can be used to diagnose bacterial, viral, and fungal corneal infection; however, it requires experienced technicians, it is affected by patient’s compliance, and image acquisition usually requires more than 10 min to scan all the cornea layers [24,25].

In this scenario, anterior segment optical coherence tomography (AS–OCT) has been successfully used to study IK because of its capability to assess the depth and extension of corneal ulceration, the infiltrate, and the haze [19,26,27,28,29]. Further, it can be used to monitor the IK and the response to treatment, especially in cases where necrotic tissue might obscure the view of the underlying tissue at the slit lamp [19]. Modern machines can acquire tomographic and keratometric images of the cornea that are particularly useful in refractive surgery [30]. Indeed, AS–OCT can help diagnose and quantify some postsurgical complications such as interface fluid syndrome, flap displacement, or epithelial ingrowth [30,31,32,33]. Reinstein et al. have also demonstrated the utility of epithelial maps for keratoconus screening, post–surgery ectasia detection, and in hyperopic patients to avoid apical syndrome following primary hyperopic treatment [34].

Although many authors have evaluated the utility of AS–OCT in different corneal diseases and corneal infections, little is known about its use in IK after refractive surgery [4,5,8,19,30,31,32,33,34,35,36]. The OCT features of an infection developing after LASIK and KLEx may considerably differ from the classical pattern of presentation, as it usually originates in the surgical interface and not through an epithelial disruption [7,37,38]. The slit–lamp clinical examination might not be able to clearly show the severity of the corneal involvement, and surgeons might decide to lift the flap because of the lack of objective parameters to assess the keratitis. This procedure is important in interface involvement but does not offer advantages if the infection is confined to the flap or there is a full–thickness epithelial defect [1]. Further, it has been demonstrated that most IK occurring after refractive surgery resolves with topical medical treatment, and only a few cases might need flap lifting and irrigation [1,39].

To our knowledge, the utility of AS–OCT in assessing the surgical interface in infectious keratitis after keratorefractive surgery has not been evaluated by previous studies. We, therefore, reviewed the OCT features of four cases of infectious keratitis occurring after LASIK and KLEx that have been studied with AS–OCT. We aimed to evaluate the efficacy and utility of AS–OCT in assessing corneal damage and surgical interface involvement in IK occurring after refractive surgery.

## 2. Material and Methods

This was a retrospective longitudinal study conducted at Siena Eye Laser (Poggibonsi, Italy). The clinical records of patients developing an infection after LASIK and KLEx were retrospectively reviewed. The inclusion criteria were diagnosis of infectious keratitis following refractive surgery, AS–OCT acquired at baseline and follow–up after treatment, and the availability of antibiotic treatment records. The diagnosis was based on slit–lamp examination (corneal infiltrates compatible with infection, hyperemia), subjective symptoms (pain, blurred vision), and culture according to previously validated criteria [6]. IK was classified as culture–proven infectious keratitis, probable culture–negative infectious keratitis, and non–infectious keratitis based on microbiological results. In case of negative cultures, the diagnosis was performed by an experienced doctor (A.L.) according to the clinical examination. In all diagnosed cases, topical treatment was started according to our standard protocol for IK with 0.5% moxiflocxacin, 1.5% fortified tobramycin, and 0.6% iodopovidone drops. Topical steroids (hydrocortisone 0.335% eye drops) were used to control the inflammatory burden and reduce the risk of corneal scarring. Written informed consent was obtained from all included subjects. This study was conducted in accordance with the Declaration of Helsinki and approved by the Institutional Review Board of Siena Eye Laser (protocol code 11/2024, 8 January 2024).

### Image Acquisition and Analysis

**Figure 1:**
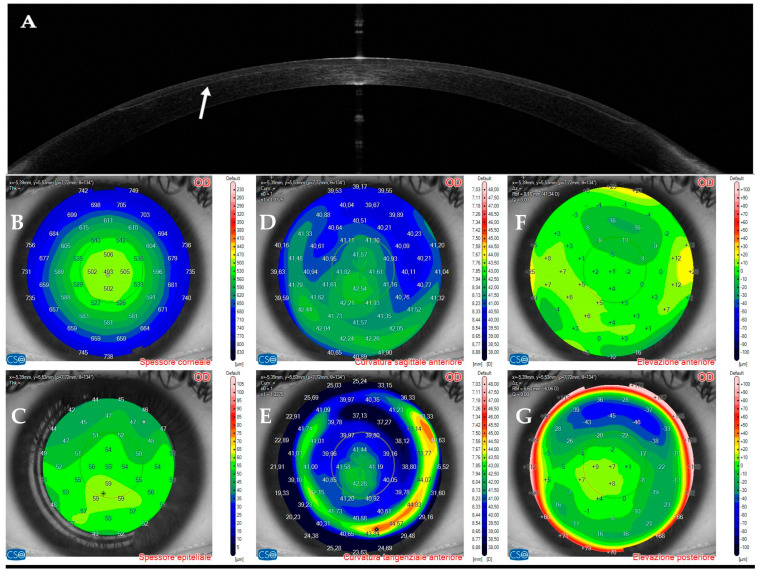
Anterior segment optical coherence tomography and topography sections (AS–OCT) 1 day after laser in situ keratomileusis (LASIK) surgery for myopia correction in a healthy patient. (**A**): AS–OCT tomography showing a regular corneal flap with a hyperdense regular line (arrow) demarcating the surgical interface. (**B**,**C**): Corneal and epithelial thickness. (**D**,**E**): sagittal and tangential anterior curvatures. (**F**,**G**): Anterior and posterior elevation.

AS–OCT MS–39 (CSO, Florence, Italy) was used to acquire tomographic and keratometric images of the corneas at baseline and follow–up. MS–39 is a tomographer that combines SD–OCT–based anterior segment tomography and Placido–disk corneal topography. Further, it creates a corneal epithelial map of 8 mm in diameter (Figure 1). It uses an 840 nm superluminescent light source to acquire corneal images with an axial resolution of 3.5 nm, a transverse resolution of 35 nm, and a depth of 7.5 mm [40]. After calibration, an experienced technician acquired three consecutive scans of patients’ eyes under the supervision of an experienced ophthalmologist (A.L.). Automatic release mode was used to acquire 16 radial scans of the cornea. Built–in software performed the biometric measures. The best representative AS–OCT image of the corneal infection was selected by two experienced ophthalmologists (A.L. and G.D.B.). Corneal thickness was assessed with the built–in software of the machine. No statistical analysis was performed because of the small number of patients included in this case series.

## 3. Results

We retrospectively reviewed refractive procedures performed at our surgical center, Siena Eye Laser. Among 22,756 eyes of 13,564 patients, we included four subjects diagnosed with infectious keratitis. Three subjects were female (75%), the mean age was 34.2 ± 2.7 years, two underwent LASIK (50%), and two underwent KLEx surgery (50%). Microbiology cultures were negative for all patients. Diagnosis of IK was performed by an experienced surgeon (A.L.) according to the clinical features, the OCT images, and the response to topical antibiotic treatment.

### 3.1. Patient #1

**Figure 2:**
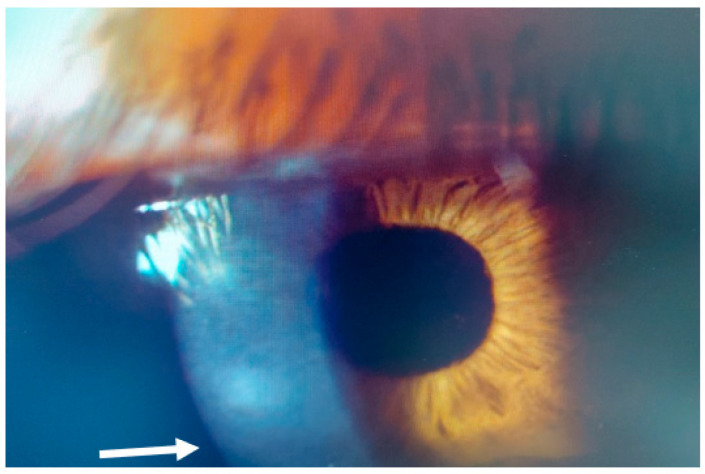
Patient #1 slit–lamp image. Right eye, corneal infiltrate (arrow) three days after laser in situ keratomileusis (LASIK).

**Figure 3:**
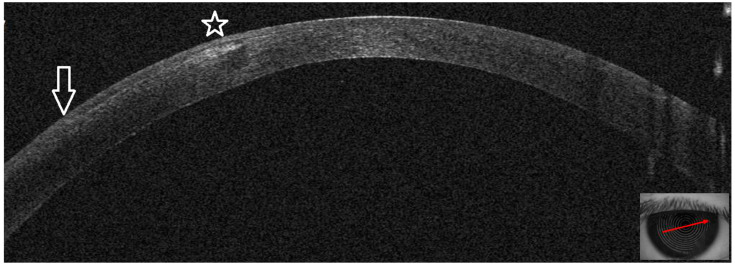
Patient #1 right eye anterior segment optical coherence tomography section (AS–OCT). Right eye, corneal infiltrate three days after LASIK. The arrow indicates the sidecut of the LASIK flap; the star is above the hyper–reflective interface infiltrate, causing dark shadowing. There is no epithelial defect.

This 36–year–old woman developed an infiltrate in the right eye three days after bilateral femtosecond LASIK for myopia correction. At presentation, slit–lamp examination showed a round paracentral infiltrate (Figure 2); the infiltrate involved the flap, but it was difficult to assess the depth and the interface involvement. AS–OCT revealed a hyper–reflective area affecting the surgical interface and the anterior stroma, causing posterior shadowing; the overlying flap and epithelium were not damaged (Figure 3).

**Figure 4:**
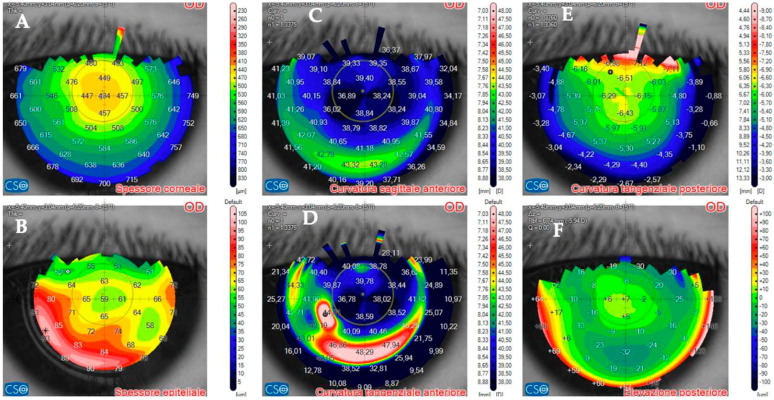
Patient #1 anterior segment optical coherence tomography (AS–OCT) corneal infiltrate three days after LASIK. (**A**): corneal thickness, modified by LASIK treatment. (**B**): epithelial thickness, showing epithelial oedema surrounding the infiltrate. (**C**,**D**): anterior tangential curvature, evidencing a locally increased curvature corresponding to the infiltrate. (**E**,**F**): posterior tangential curvature and posterior elevation modified after LASIK treatment.

On OCT tomography, the anterior tangential map showed an area of increased curvature corresponding to the infiltrate and the epithelial map revealed moderate thickening peripherally to the infiltrate (Figure 4). An experienced surgeon (A.L.) diagnosed a corneal infection, and corneal cultures were taken. The AS–OCT section showed the presence of an infiltrate in the interface, confirming that the infection was localized to that corneal area. No deep stromal involvement or anterior chamber inflammation was seen. Stromal thickness was 433 microns (Table 1). Therefore, we decided to apply our standard protocol for IK with an empiric intense antibiotic topical treatment.

**Figure 5:**
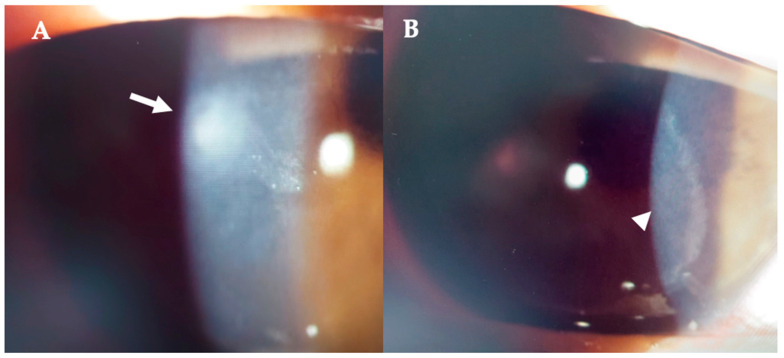
Patient #1 right eye slit–lamp image. Corneal infiltrate five days after LASIK. (**A**)**:** Corneal infiltrate after three days of topical antibiotics (arrow). (**B**): ring–shaped pseudo–infiltrate (arrowhead) surrounding an area of corneal oedema (arrowhead).

**Figure 6:**
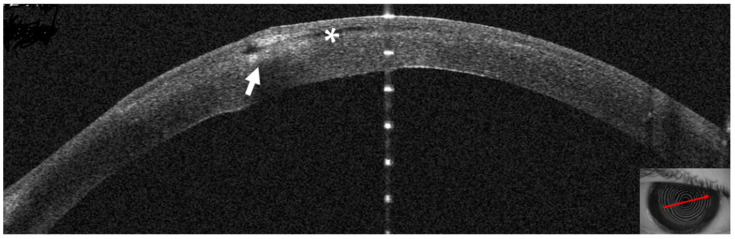
Patient #1 right eye anterior segment optical coherence tomography section (AS–OCT). Corneal infiltrate five days after LASIK. The infiltrate shown in Figure two has grown and is now divided in two by a dark space (asterisk), corresponding to a fluid layer, which extends to the center of the surgical interface. The whole stroma is thickened by diffuse oedema (arrow).

Topical treatment with 0.5% moxiflocxacin, 1.5% fortified tobramycin, and 0.6% iodopovidone was started. Two days after the presentation, the patient complained of blurred vision; at the slit lamp, the infiltrate appeared denser and surrounded by a ring (Figure 5A,B). On AS–OCT, the infiltrate was thicker, localized stromal oedema was present, and the temporal interface was separated by a layer of fluid, causing the ring effect at the slit lamp (Figure 6). Corneal thickness had increased to 567 microns (Table 1).

**Figure 7:**
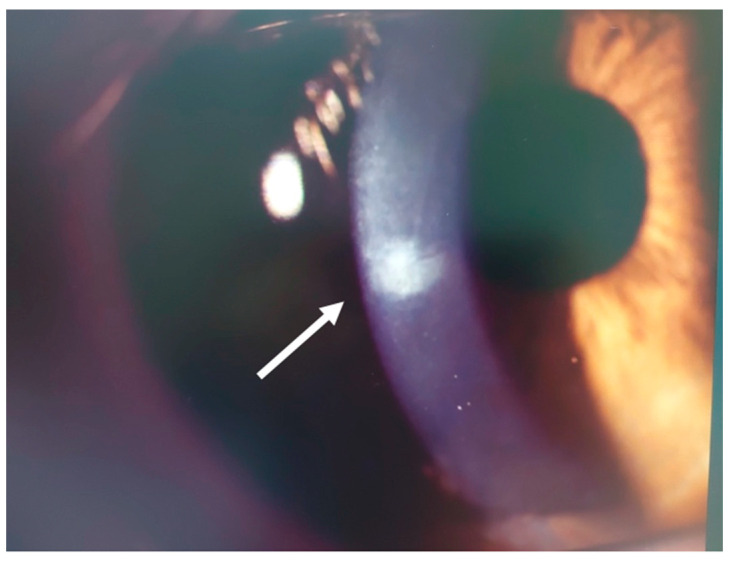
Patient #1 right eye slit–lamp image. Corneal infiltrate (arrow) 12 days after LASIK. The infiltrate has healed, and there is a mild residual.

**Figure 8:**
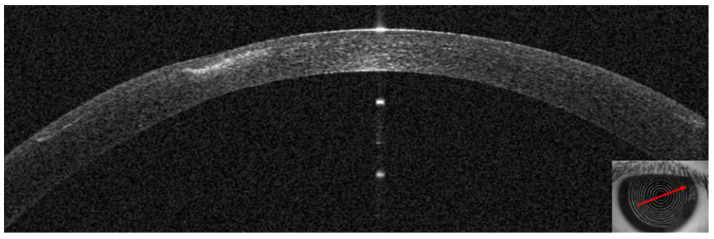
Patient #1 anterior segment optical coherence tomography section (AS–OCT). Corneal infiltrate 12 days after LASIK. The AS–OCT image shows a hyperdense area, overlying stromal thinning, and no fluid layer as signs of improvement of the infection.

**Figure 9:**
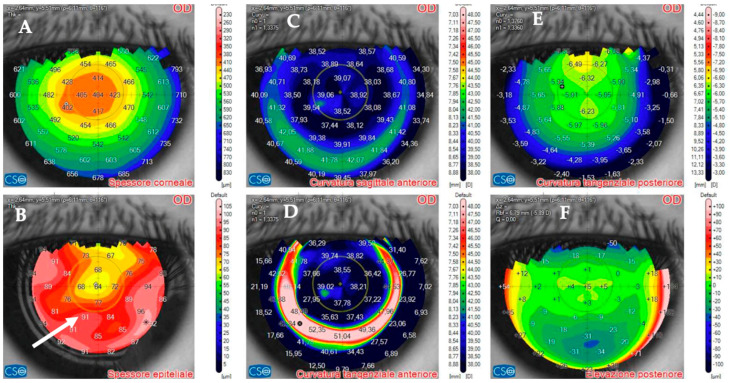
Patient #1 anterior segment optical coherence tomography (AS–OCT) of the right eye corneal infection 12 days after LASIK at AS–OCT tomography. The overall corneal thickness has decreased by 30 µm (**A**). The epithelium has instead thickened as a compensatory response to the infection (arrow, (**B**)). The corneal anterior curvature has returned to almost complete regularity (**C**,**D**). Corneal posterior tangential curvature and posterior elevation have recovered. The images show normal post–LASIK appearance of the maps (**E**,**F**).

Twelve days after presentation, symptoms had resolved, and the infiltrate had a cicatricial aspect with neat margins and no surrounding oedema (Figure 7). On AS–OCT, a hyperdense area was still present, with overlying stromal thinning (Figure 8) and a corneal thickness of 398 microns (Table 1). OCT tomography showed epithelial thickening and mild residual irregular elevation on anterior tangential curvature (Figure 9).

Topical corticosteroids (hydrocortisone 0.335% eye drops) were started to control the inflammation and reduce the risk of corneal scarring.

**Figure 10:**
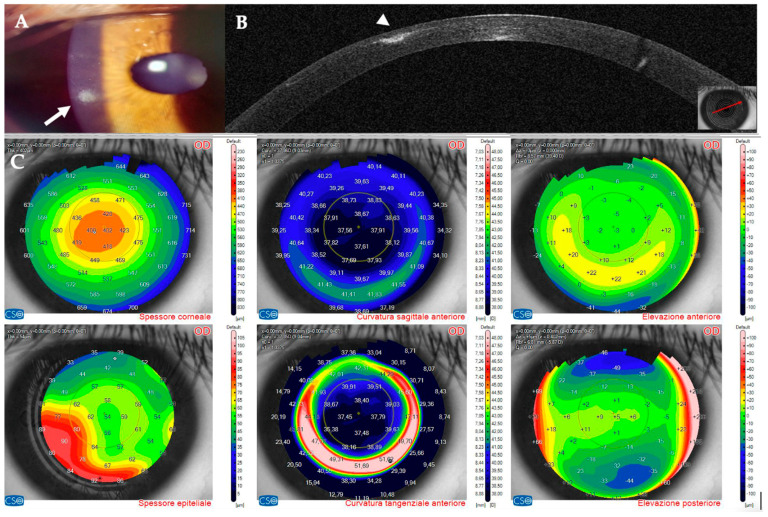
Patient #1 right eye multimodal imaging one month after infectious keratitis. (**A**): corneal infiltrate (arrow). The scar has been partially reabsorbed. (**B**): anterior segment optical coherence tomography section (AS–OCT). Stromal reformation and epithelial remodeling have filled the previously thinned area (arrowhead). (**C**): AS–OCT tomography showing that the overall corneal thickness has recovered to the postoperative value. The epithelium now has a normal thickness, except for the area surrounding the scar.

At one month, the infiltrate had regressed to a small nubecola (Figure 10A), the corneal thickness had recovered to 407 microns (Figure 10B, Table 1), and tomography maps showed a regular anterior tangential curvature with almost normal epithelial thickness (Figure 10C). Unaided visual acuity was 20/16.

### 3.2. Patient #2

This 35–year–old woman developed an infiltrate in the right eye 18 days after bilateral femtosecond LASIK for myopia.

**Figure 11:**
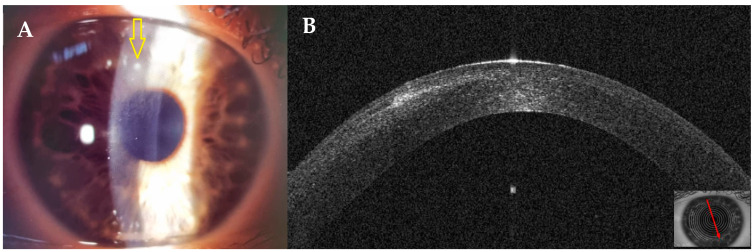
Patient #2 right eye multimodal imaging of corneal infectious keratitis after LASIK. (**A**): Right eye, small round superior infiltrate surrounded by mild oedema (yellow arrow) 18 days after LASIK. (**B**): right eye anterior segment optical coherence tomography (AS–OCT) section showing a thin hyper–reflective area in the surgical interface with overlying intact epithelium.

At presentation, a small round superior infiltrate surrounded by mild oedema was evident at the slit lamp (Figure 11A). The AS–OCT revealed a thin hyper–reflective area in the surgical interface; the overlying flap and epithelium were not damaged, and the interface was slightly hyperdense (Figure 11B). Stromal thickness was 433 microns (Table 1). Topical treatment was administered as in patient #1.

**Figure 12:**
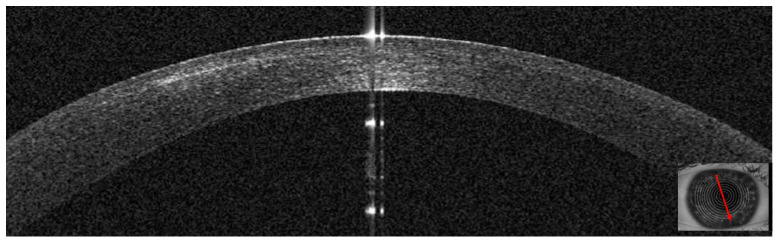
Patient #2 right anterior segment optical coherence tomography (AS–OCT) section. The image shows the resolution of the interface infiltrate 3 days after antibiotic treatment was started.

Three days after presentation, the infiltrate had resolved; only slight interface inflammation was evident (Figure 12). Corneal thickness had improved to 424 microns (Table 1). Unaided visual acuity was 20/20. Topical treatment with hydrocortisone 0.335% was started. Twenty days after presentation, AS–OCT images and corneal thickness remained stable (Table 1).

### 3.3. Patient #3

**Figure 13:**
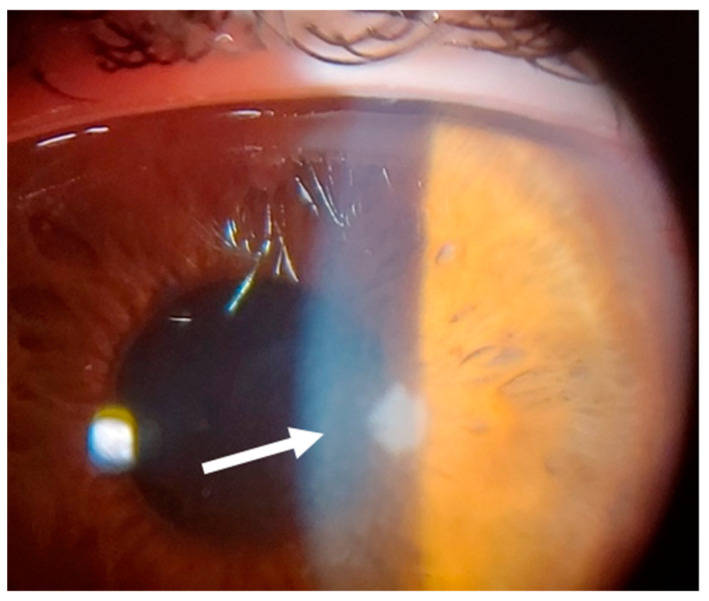
Patient #3 left eye slit–lamp image. Left eye corneal infiltrate (arrow) 24 days after keratorefractive lenticule extraction (KLEx).

**Figure 14:**
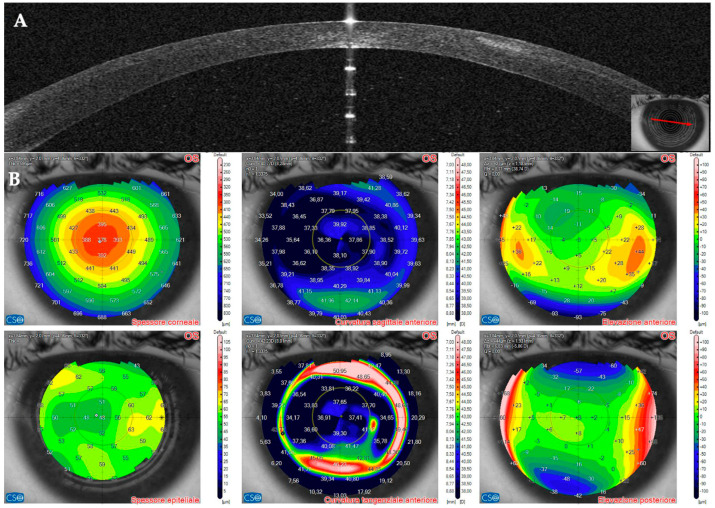
Patient #3 multimodal imaging of the left eye. (**A**): Left eye anterior segment optical coherence tomography (AS–OCT) section showing a localized hyper–dense infiltration in the interface. (**B**): AS–OCT tomography showing irregularity of the anterior tangential map.

This 34–year–old woman developed an infiltrate in the left eye 24 days after bilateral KLEx for myopia. At the slit lamp, a paracentral, well–defined infiltrate was visible (Figure 13), corresponding on AS–OCT to a localized hyper–density in the interface (Figure 14A) and causing a slight irregularity on anterior tangential tomography (Figure 14B). Corneal thickness was 378 microns (Table 1). Topical treatment was started as in patient #1.

**Figure 15:**
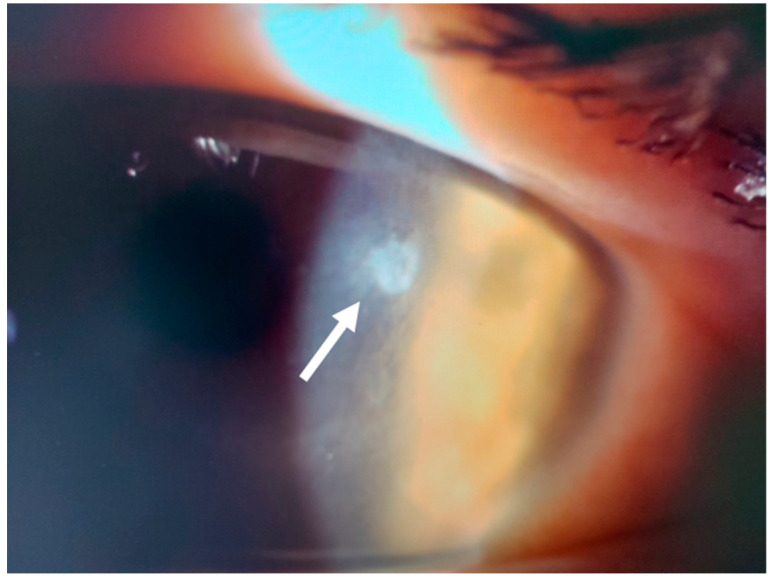
Patient #3 left eye slit–lamp image. Left eye corneal infiltrate (arrow) 31 days after keratorefractive lenticule extraction (KLEx).

Seven days after presentation, the infiltrate was less defined and was associated with interface inflammation and oedema (Figure 15).

**Figure 16:**
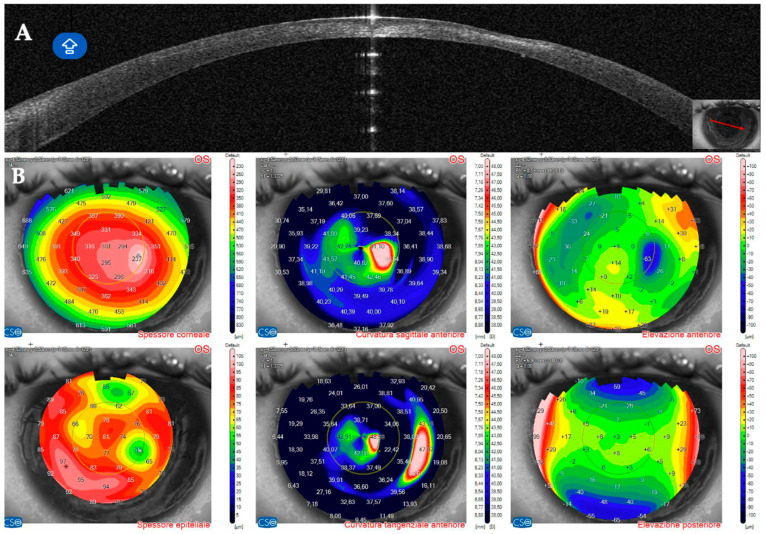
Patient #3 anterior segment optical coherence tomography (AS–OCT) section 31 days after keratorefractive lenticule extraction (KLEx). (**A**): Left eye AS_OCT section showing epithelial and stromal thinning at the level of the corneal infiltrate. (**B**): AS–OCT tomography showing irregularity of the anterior tangential and sagittal maps. The epithelial map shows an area of thinning matching the area of the corneal infection, surrounded by a thicker epithelium.

On AS–OCT sections and tomography, a generalized corneal thinning was evident, more pronounced in the lesion area, locally thick at only 237 µm (Table 1), with global epithelial oedema sparing the lesion area (Figure 16A,B). Topical corticosteroids (hydrocortisone 0.335% eye drops) were started to control the inflammatory burden.

**Figure 17:**
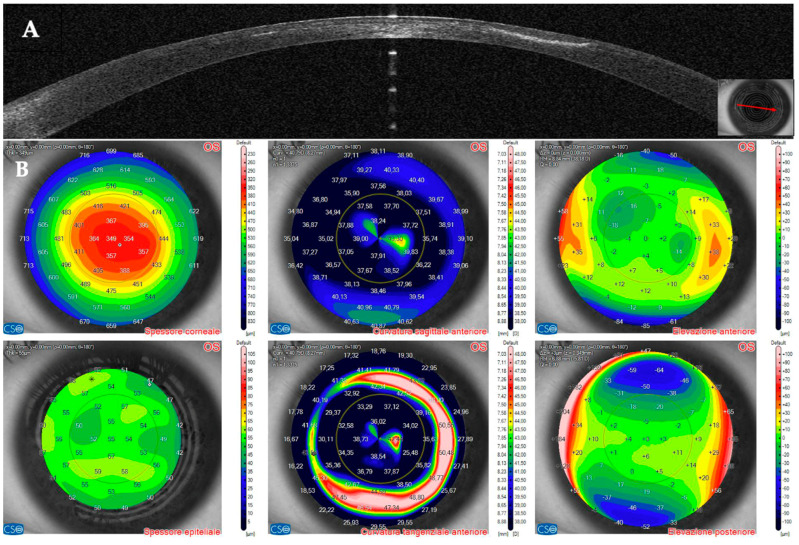
Patient #3 left eye anterior segment optical coherence tomography (AS–OCT) section. (**A**): Left eye AS–OCT section showing resolution of the corneal infiltrate at eight weeks. Corneal thickness has recovered to pre–infection values, and the image shows the epithelial compensation. (**B**): AS–OCT tomography showing a more regular appearance of the anterior tangential and sagittal maps. The epithelial map shows normal epithelial thickness.

Two months later, the infectious process resolved, and the cornea recovered to post–operative thickness, with a compensatory reformation of the stroma and epithelium (Figure 17A,B). Corneal thickness had improved to 346 microns (Table 1). Visual acuity was 20/25 with +3 × 83°.

### 3.4. Patient #4

**Figure 18:**
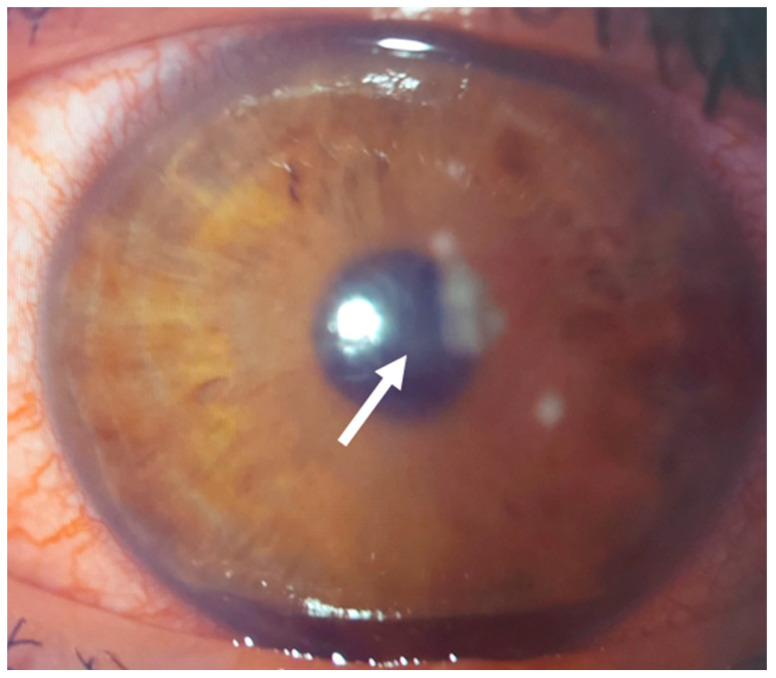
Patient #4 slit–lamp image. Left eye, multiple corneal infiltrates (arrow) two days after KLEx.

**Figure 19:**
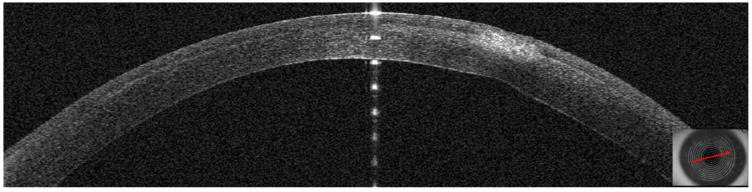
Patient #4 left eye anterior segment optical coherence tomography (AS–OCT) section. Left eye corneal infection two days after KLEx. On AS–OCT, one of the infiltrates is centered in the surgical interface, expanding externally, with a thin hyper–reflective line corresponding to the inflamed interface.

Two days after bilateral KLEx for myopia, this 31–year–old man developed multiple infiltrates in the surgical interface, causing interface inflammation (Figure 18 and Figure 19). Visual acuity had dropped to 20/30 from 20/20 on post–operative day one. Corneal thickness was 497 microns. Topical treatment with antibiotics was started.

**Figure 20:**
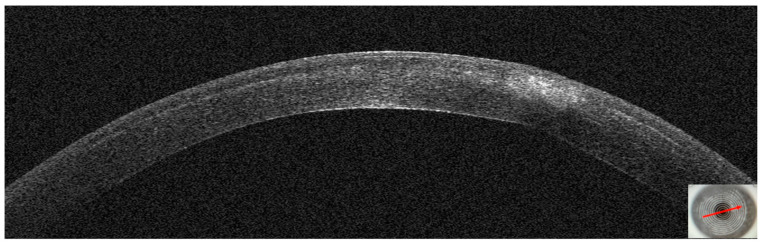
Patient #4 left eye anterior segment optical coherence tomography (AS–OCT) section 5 days after corneal infection. On AS–OCT, the infiltrate is less dense, but there is more surrounding oedema.

Five days after the presentation, the infiltrates looked less dense, but there was more surrounding oedema. Corneal thickness had increased to 597 microns (Figure 20) as a result of corneal inflammation. The patient was started on topical steroids.

**Figure 21:**
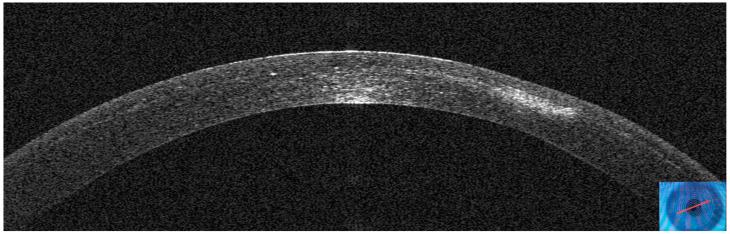
Patient #4 left eye anterior segment optical coherence tomography (AS–OCT) section 10 days after corneal infection. On AS–OCT, the infiltrate has a cicatricial aspect, and corneal oedema and inflammation have resolved.

At ten days, visual acuity had improved to 20/20, and AS–OCT showed resolution of corneal oedema. The infiltrate looked thinner and had a cicatricial aspect (Figure 21). Corneal thickness had improved to 443 microns.

## 4. Discussion

In the present case series, we demonstrated that AS–OCT is a useful tool for assessing infectious keratitis after refractive surgery. The device can help identify interface inflammation, stromal oedema, fluid accumulation, changes in stromal and epithelial thickness, and changes in corneal curvature and elevation. Furthermore, the AS–OCT is also important for evaluating the response to the topical treatment and the regularization of the corneal curvature.

Infectious keratitis following refractive surgery is a rare event that needs prompt recognition and management to avoid severe complications and sequelae, such as corneal scarring and blindness [1]. The routine examination with a slit lamp has some limitations, mostly related to the physical features of the light source and the difficulty in evaluating the infection depth and the associated oedema [26]. This is particularly true when necrotic tissue is present, which might obscure the view of the underlying corneal layers [19]. In this scenario, AS–OCT has been demonstrated to be a useful tool to assess patients and guide treatment [26,29]. AS–OCT during primary infectious keratitis can differentiate scars from infiltrates, both hyper–reflective, the latter presenting with an overlying epithelial defect or “opaque” epithelium, and with rounder and less defined margins [26,27,29,33,41]. Soliman et al. used AS–OCT to assess patients with microbial keratitis and identified ten image patterns common to fungal and bacterial infections [27]. Furthermore, they also found that localized and diffuse necrotic cystic spaces were specific to fungal infections, a finding that might guide clinicians in diagnosis and treating fungal keratitis. AS–OCT can also measure the corneal thickness and estimate the risk of corneal perforation and the efficacy of the topical treatment [8]. In a prospective study, Konstantopoulos et al. used AS–OCT to monitor bacterial keratitis in clinical practice by assessing corneal thickness (CT) and infiltrate thickness (IT) [28]. Their work shows that repeated AS–OCT scans can be used to assess corneal inflammation and response to treatment, especially in an early stage of the infection when successful treatment results in a reduction of oedema, CT, and IT. Moreover, AS–OCT can also be used to assess anterior chamber inflammation, especially in case of corneal opacity caused by the corneal infection [42,43,44]. As demonstrated by Liu et al. [36] in a patient with IK after KLEx, the presence of anterior chamber reaction with/without endothelial plaques may suggest worsening of corneal infection and might require more aggressive treatment, such as interface irrigation or cap amputation.

**Figure 22:**
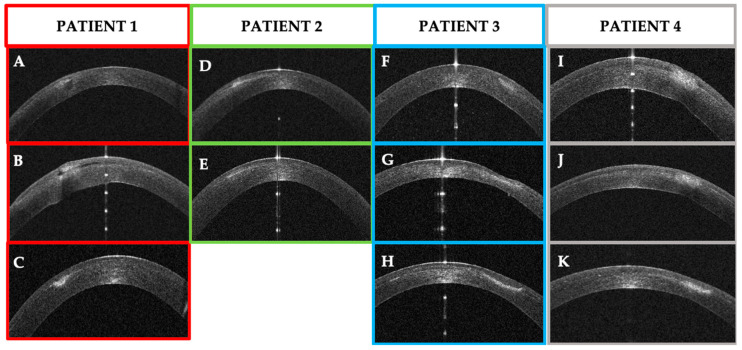
Patients’ corneal changes on anterior segment optical coherence tomography (AS–OCT) before and after treatment. (**A**–**C**): Patient #1 AS–OCT images of the corneal infection at baseline (**A**), day 5th (**B**) and day 30th after treatment. D–E: Patient #2 AS–OCT images of corneal infiltrate at baseline (**D**) and at day 20th (**E**). F–G–H: Patient #3 AS–OCT images of corneal infection at baseline (**F**), day 7th (**G**), and after resolution at day 56th (**H**). (**I**–**K**): Patient #4 AS–OCT images of corneal infection at baseline (**I**), day 3rd (**J**), and day 10th.

In our cases, where the infection started at the surgical interface, the epithelium was not altered at presentation, and the AS–OCT showed stromal and epithelial oedema in more advanced phases. Further, we could also assess the correct depth of the infection and the presence or lack of stromal lysis (Figure 22). In one case, we also saw a late epithelial defect that healed once the infection settled down. In our study, the AS–OCT was able to show the efficacy of the treatment and improvement in the appearance of the corneal infection (Figure 21). Further, in all four cases, we did not detect any inflammation in the anterior chamber, confirming that the keratitis was limited to the corneal epithelium/stroma without infective or inflammatory involvement of the internal parts of the eye.

Modern AS–OCT machines combine tomography and topography modules to study corneal curvatures and can provide epithelial maps [34]. In refractive surgery, the procedure is usually performed in young, healthy patients with high expectations, and a corneal infection can represent a severe complication even if promptly treated and resolved [1]. In this scenario, when a corneal infection occurs after refractive surgery, it is important to study the corneal curvature changes to predict the final refractive outcome [2,45]. In a 2008 survey from the American Society of Cataract and Refractive Surgery (ASCRS), 19 IK were reported on 20,941 keratorefractive procedures, and three patients (15.7%) had a final visual acuity of 20/40 or worse, with two subjects requiring corneal transplantation to restore vision [45]. Once an infection has occurred and has altered the corneal tissues, the restoration of the corneal shape and the vision relies on epithelial remodeling to compensate for the corneal damage [34,46]. In the early stages of corneal infections, epithelial mapping with AS–OCT can show epithelial thinning in the infected corneal area surrounded by thickened epithelium. After infection resolution, some patients developed stromal thinning and scarring, with compensatory overlying thickened epithelium compensating for the stromal loss [34,47]. 

In our study, we used AS–OCT to quantify post–infectious scars and irregularities. Patients one, two, and three demonstrated altered corneal tomographies during the early stages of the infections. After the resolution of the IK, epithelium thickened to balance the stromal loss, especially in patient three, where the stromal lysis was more severe. Regularization of anterior sagittal and tangential corneal curvature correlated with restoration of the visual acuity, confirming the key role of the epithelium in compensating for tissue loss and corneal irregularity.

*Acanthamoeba keratitis* (AK) is a potentially sight–threatening infection caused by a free–living protozoan that colonizes soil and water, in particular tap water [48]. The diagnosis is challenging because it can present with specific signs and symptoms, and prompt diagnosis is important to prevent corneal scarring and blindness [23,49]. AS–OCT utility has been described in AK, and studies have identified some recurrent specific patterns of the infection [41,50]. In a cross–sectional study, Oliveira et al. identified AS–OCT morphological features of early AK infections characterized by the presence of hyperreflective stromal lesions and stromal oedema [41]. In a prospective study, Yamazaki et al. used AS–OCT to detect Acanthamoeba cysts as highly reflective round particles and radial keratoneuritis as highly reflective bands [50]. In our case #1, a ring pseudo–infiltrate was evident at the slit lamp, but AS–OCT showed a corneal infiltrate at the edge of the fluid collection in the surgical interface in the absence of all the other amoebic characteristics.

The use of AS–OCT in infectious keratitis after refractive surgery has been the subject of few articles, reporting interface infiltrates, flap perforation, and opaque epithelium [37,38]; in more severe cases, pronounced oedema and endothelial plaques can be present [36]. Around the infiltrate, the surgical interface appeared denser in one case of presumed bacterial keratitis in KLEx [38]. In the case of mycobacterial keratitis after LASIK, the AS–OCT might differentiate epithelial from stromal opacity [37]. After healing, a hyper–reflective interface was observed, corresponding to clinical haze [5]. In our study, the AS–OCT allows us to clearly localize the corneal infiltrate into the surgical interface as a hyperdense lesion. In case one, the presence of fluid showed a higher level of inflammation, and its resolution after treatment was considered a clear sign of improvement and response to topical antibiotics.

DLK is an uncommon complication of LASIK [51] and KLEx [52]. It is caused by an immune reaction to chemical and biological substances introduced in the interface (including products of sterilization, glove powder, and ink) [35] and to excess energy in the femtosecond laser energy [53]. In more severe cases, it can lead to stromal reabsorption, first compensated by epithelial thickening, then by stromal reformation [51]. Although it usually affects both eyes in case of simultaneous bilateral surgery, unilateral keratitis is described in the literature [54], and it is important to differentiate DLK from a corneal infection. Indeed, IK, after refractive surgery, can present with interface inflammation and fluid accumulation [45]. In our patient #1, there was a fluid layer in the surgical interface associated with a focal infiltrate that guided us in the diagnosis of IK rather than DLK. In our patient #3, a pattern of stromal reabsorption involving the whole surgical interface was observed during the healing phase. In this patient, the stromal alteration pattern might have been confused with those of DLK, but the lack of cellular infiltration typical of post–LASIK DLK [51], as reported by Reinstein et al. [52], helped us in the differential diagnosis between IK and DLK.

This study has some limitations. First, we included only four cases of infectious keratitis. However, we know that IK is a rare event after corneal refractive procedures, and this case–series study may represent a first step to further investigate the utility of AS–OCT in refractive surgery. Second, we did not identify the microorganisms that caused the corneal infection. As previously explained, most of the IK in refractive surgery resolves with medical treatment, and we did not find it necessary to lift the flap and irrigate the cornea. A slit–lamp assessment was made by an experienced ophthalmologist, and corneal lesions quickly improved after antibiotic treatment was started, confirming the diagnosis of infectious keratitis.

## 5. Conclusions

In this study, we demonstrated that AS–OCT can be a useful tool for assessing corneal infections after refractive surgeries. In four cases of IK, AS–OCT provided an excellent picture of the infiltrate and the associated abnormalities, such as stromal oedema, interface inflammation, stromal reabsorption and reformation, epithelial changes, and fluid layers. Further, since all the cultures were negative, we used the AS–OCT to assess the antibiotic treatment efficacy and the resolution of the corneal infections. To our knowledge, this study outlines for the first time the importance of surgical interface evaluation with AS–OCT in the assessment and treatment of IK after refractive surgery. Prospective studies are needed to confirm our preliminary results.

## Figures and Tables

**Table 1:** Corneal thickness changes before (baseline) and after treatment of infectious keratitis.

Patient	Baseline	Follow–Up 1	Follow–Up 2	Follow–Up 3
Patient #1 thickness (days)	433 microns	567 microns (5)	398 microns (12)	407 microns (30)
Patient #4 thickness (days)	433 microns	424 microns (21)	417 microns (20)	
Patient #3 thickness (days)	233 microns	346 microns (56)		
Patient #4 thickness (days)	497 microns	597 microns (3)	443 microns (10)	

## Data Availability

The raw data supporting the conclusions of this article will be made available by the authors upon request.
